# A Fringe Phase Extraction Method Based on Neural Network

**DOI:** 10.3390/s21051664

**Published:** 2021-02-28

**Authors:** Wenxin Hu, Hong Miao, Keyu Yan, Yu Fu

**Affiliations:** 1 Shenzhen Key Laboratory of Intelligent Optical Measurement and Detection, College of Physics and Optoelectronic Engineering, Shenzhen University, 3688 Nanhai Avenue, Shenzhen 518060, China; huwenxin@szu.edu.cn (W.H.); yankeyu@szu.edu.cn (K.Y.); 2 CAS Key Laboratory of Mechanical Behavior and Design of Materials, Department of Modern Mechanics, University of Science and Technology of China, Hefei 230027, China; miaohong@ustc.edu.cn

**Keywords:** phase extraction, U-net neural network, warped phase map, fringe pattern

## Abstract

In optical metrology, the output is usually in the form of a fringe pattern, from which a phase map can be generated and phase information can be converted into the desired parameters. This paper proposes an end-to-end method of fringe phase extraction based on the neural network. This method uses the U-net neural network to directly learn the correspondence between the gray level of a fringe pattern and the wrapped phase map, which is simpler than the exist deep learning methods. The results of simulation and experimental fringe patterns verify the accuracy and the robustness of this method. While it yields the same accuracy, the proposed method features easier operation and a simpler principle than the traditional phase-shifting method and has a faster speed than wavelet transform method.

## 1. Introduction

Optical metrology has been widely used in various areas, such as 3D sensing, machine vision, intelligent robot control, industry monitoring, and dressmaking. In optical metrology, the output is usually in the form of a fringe pattern, from which a phase map can be determined. Once the phase map has been obtained, it can be converted into the desired parameters, such as the shape of the object, and in-plane or out-of-plane deformation. For instance, the fringe projection technique [1,2,3,4] is often used to measure the 3D-profile of objects [2]. When the fringe pattern is projected on a measured free surface, the phase of the fringe pattern is modulated by the height distribution of the object. A method to extract the phase map from the deformed fringe pattern is thus needed.

Thus far, many methods for phase calculation have been developed, including temporal phase-shifting [4,5], spatial phase-shifting [6,7], and Fourier transform [8]. The phase-shifting is a pointwise technique and it is sensitive to noise such as CCD random noise, environmental vibration, air disturbance, etc. The temporal phase-shifting method requires four images in one stage, which is unsuitable for real-time measurement, and the spatial phase-shifting method requires a complex optical path. The Fourier transform technique, on the contrary, is a global transform method that is hence more tolerant to noise. However, as the transform is global, an accurate frequency band containing effective information of the measured object needs to be determined to avoid large calculation errors. Some improvements have been proposed to overcome the shortcoming of the simple Fourier transform method. A windowed Fourier ridges algorithm [9,10,11] and a windowed Fourier filtering algorithm have been proposed to achieve a low standard deviation for local frequencies and phase distributions in fringe pattern analysis. Morlet wavelet transform has also been used for phase extraction on different types of fringe patterns [12,13,14].

In this research, we propose a fringe phase extraction method based on the neural network. As an important part of machine learning, neural networks have been widely used in various fields, such as object recognition [15,16,17,18], object segmentation [19], and speech recognition [20,21]. This method has also been introduced to optical measurement. Liu et al. employed the backpropagation (BP) [22,23] artificial neural network to directly build a nonlinear mapping relationship between the gray-gradient of speckle images before and after deformation, and sub-pixel displacement in the digital image correlation method. This method avoids the least squares analytical optimal solution of the correlation coefficient. Horisaki et al. have used support vector regression (SVR) to recover the image through the scattering layer [24]. This approach enables model-free sensing, where it is not necessary to know the sensing processes/models. Guan et al. have introduced a method of grating sub-division based on the radial-basis function (RBF) neural network. It converts displacement into a digital measure that is transmitted to the microprocessor of a neural network to obtain the sub-division value. This improves the accuracy of sub-division and the tracking speed of the displacement [22]. Rivenson et al. have proposed a holographic image reconstruction method based on the convolutional neural network (CNN) that can reconstruct the phase and amplitude of images of objects using only a hologram [25]. Pitkaaho et al. have employed the CNN to focus on automatic distance calculation in holographic image reconstruction [26]. Wang et al. have proposed a one-step end-to-end learning-based method for in-line holographic reconstruction that creates a network called eHoIoNet to avoid phase shifting [27]. Deep-learning based temporal phase unwrapping (DL-TPU) is introduced by Wei Yin [28], which can substantially improve the unwrapping reliability compared with multi-frequency temporal phase unwrapping (MF-TPU). These results show that challenging problems in optical metrology can be overcome through machine learning, and provide new avenues for image analysis. Shijie Feng et al. has introduced a machine-learning-based fringe analysis method, which employs two convolutional neural networks (CNN1 and CNN2) to calculate phase information [29]. For CNN2, the inputs are the fringe pattern and the background image predicted by CNN1, and the outputs are the numerator and the denominator, which are then fed into the arctangent function to calculate phase. Some improvement and simplification have also been made by them. A micro deep learning profilometry using a single network is presented for high-speed 3D surface imaging [30]. However, three fringe patterns are needed for correct phase unwarpping. Haotian Yu et al. has introduced a novel phase retrieval method based on a deep neural network called FPPnet [31]. The FPPnet only requires one single image and one single network, and this network is used to achieve prediction of output fringes in the same period and different periods. Then, the phase calculation and the phase unwrapping can be achieved by these predicted fringes. However, these methods employ neural network to obtain intermediate calculation parameters such as numerator or denominator or related fringe pattern, not directly acquiring phase information. Furthermore, a deep-learning-based approach is proposed by Sam to extract height information from single deformed fringe patterns [32]. The fully CNN is trained on a large set of simulated height maps with corresponding deformed fringe patterns, so phase results rely too much on the complexity of these simulated height maps.

In this paper, we introduce a one-step deep-learning-based method to extract the wrapped phase map directly from a single fringe pattern. This method employs the U-net neural network to directly learn the correspondence between the gray level of a fringe pattern and the wrapped phase map. Once a stable network model has been obtained, the wrapped phase map of an arbitrary fringe pattern can be output directly, thus simplifying the phase extraction further. The mathematics problem is transformed into image processing problem, developing the advantage of neural network. Meanwhile, the network can be saved and shared. More and deeper training contributes to the network generalization ability, so as to solve more complex and different fringe patterns. Besides, experimental results verified effectiveness on different fringe pattern whether coming from fringe projection profilometry or interferometer. While it yields the same accuracy, the proposed method features easier operation and a simpler principle than the traditional phase-shifting method, and it owns faster computation speed and higher accuracy than wavelet transform method. Moreover, the results of simulated and experimental fringe patterns verify the efficiency and the robustness of the proposed method.

## 2. Method

### 2.1. Principle

The U-net is an end-to-end deep neural network that takes an image of any size (fringe patterns here) as input and outputs a specified image (corresponding wrapped phase maps here). The process of forward propagation, the training method and output principle used for the neural network are described in Section 2.2, Section 2.3 and Section 2.4, respectively. As our ultimate goal is to obtain a stable network model with effective parameters, a large amount of training data, including fringe patterns and corresponding wrapped phase maps, are needed in advance. Once this stable network model has been obtained, the wrapped phase map of an arbitrary fringe pattern can be obtained directly. The neural network method was programmed in Python based on the Tensorflow framework, and run on a desktop computer equipped with an Intel i5-4460 CPU and a GeForce GTX 1080 graphics card.

### 2.2. U-Net Neural Network

The size of the input fringe pattern is 512×512 pixels and the output maintain the same size. This network features a contracting path, a transition path and an expansive path. The contracting path is used to extract features of the fringe pattern, and the expansive path is applied for converting into corresponding warped phase map. With the deepening of the contracting layers, low-dimensional features including gray gradient of every pixel are changed into high-dimensional features including the location and the local gradient. More layers of each path mean more connection parameters, so as to fit more complex non-linear mapping relationship.

The principle of the contracting path is the same as that of the CNN [33]. The contracting path includes four repeated down-sampling process. Every down-sampling contains two convolution blocks and a pooling block. The feature channel doubles every two convolution blocks, and the image dimensions reduce the half after a pooling operation because the stride is two pixels.

**Figure 1:**
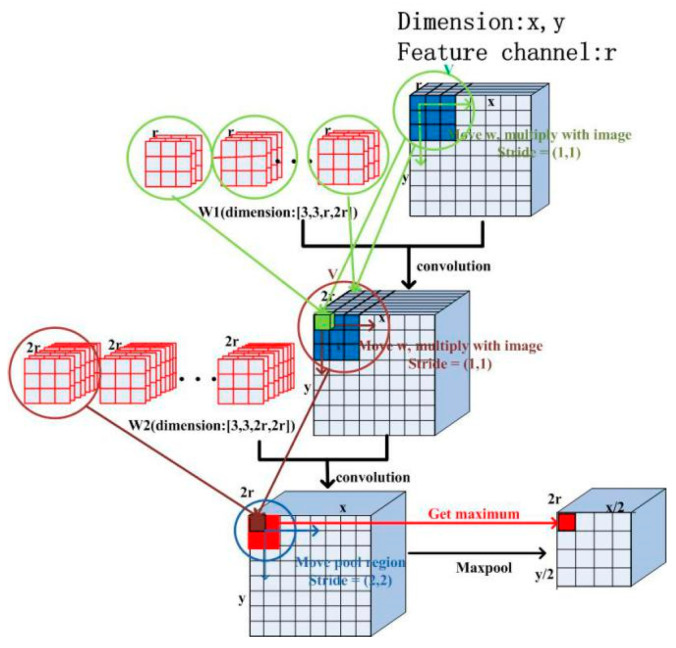
Process of down-sampling.

The down-sampling operation is illustrated in Figure 1. As Figure 1 shows, the convolution kernel shifting stride is (1, 1) along two dimensions. This means that the convolution kernels shift 1 pixel along the x and y directions each time and multiple with the image. The convolution consists of a convolution layer and an activation function, and the principle [34] can be described by Formula (1):

(1) $$v_{i,r_{2}}^{x,y}=f\left(\overset{R}{\underset{r_{1}=1}{\sum }}\overset{P-1}{\underset{p=0}{\sum }}\overset{Q-1}{\underset{q=0}{\sum }}w_{i,r_{2},r_{1}}^{p,q}v_{i-1,r_{1}}^{x+p,y+q}+b_{i,r_{2}}\right),$$

where $v_{i,r_{2}}^{x,y}$ represents the value of the output at (x,y) for the $r_{2}$-th feature channel map of the i -th layer. $v_{i-1,r_{1}}^{x+p,y+q}$ represents the value of the result at (x+p,y+q) for the $r_{1}$-th feature channel map of the (i−1) -th layer, and R is the total number of feature channel in the (i−1) -th convolution layer. $b_{i,r_{2}}$ is a common basic term for the $r_{2}$-th feature channel map of the i -th layer. $w_{i,r_{2},r_{1}}^{p,q}$ represents the weight of the convolution kernel at (p,q), and P×Q is the size of the convolution kernels in terms of pixels, which is 3×3 in all the convolution blocks of the contraction path. f represents the activation function, which uses rectified linear units (ReLUs) [33]. The principle of ReLU is described by Formula (2):

(2) f(x)=RELU(x)=max(0,x),

The pooling block employs the max-pooling method, and the region of every pooling operation is 2×2 pixels, which is intended to obtain the maximum pixel value in this small region. Additionally, the pooling stride is (2, 2) along the x and y directions so as to reduce the image size by a factor of 2.

**Figure 2:**
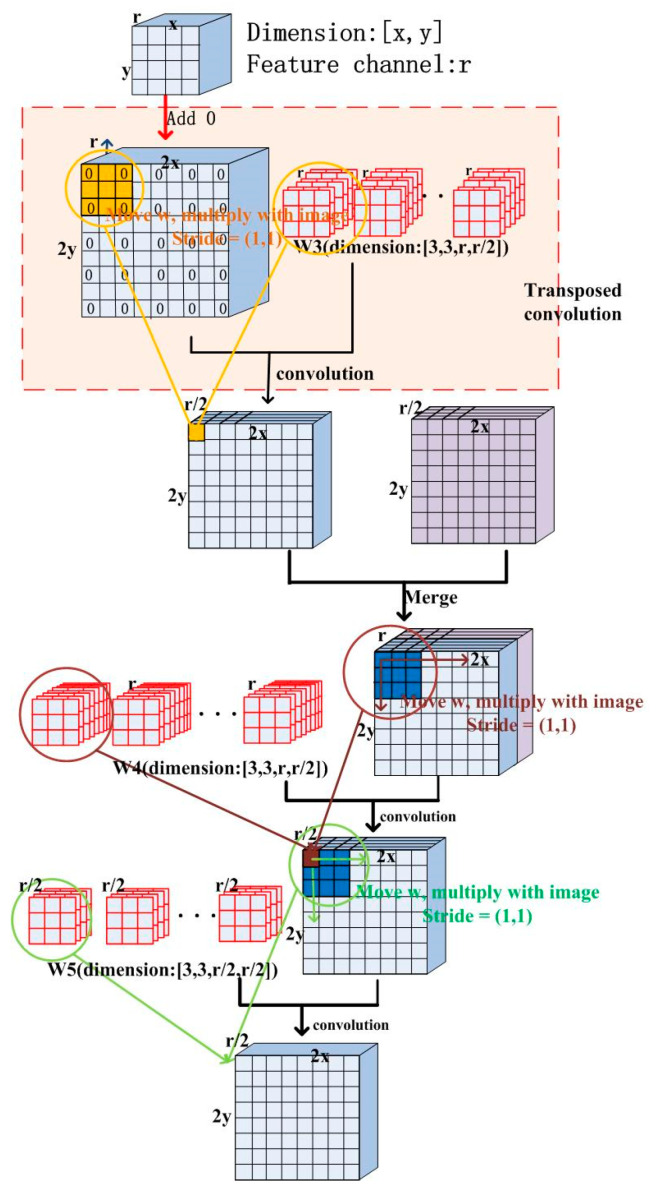
Process of up-sampling.

The expansive path includes four repeated up-sampling process, and it aims to enlarge the image and decode the convolution process. The up-sampling operation including a transposed convolution block, a merge block and two convolution blocks is illustrated in Figure 2. This operation doubles the size of the image and reduces the number of feature channel by half.

The transposed convolution operation is identical to the convolution operation, but it enlarges the image from the previous block. Some zero-value pixels between neighboring image pixels are inserted, and a convolution operation on the up-sampled image is employed. The stride of the transported convolution layer is 2×2 pixels, which means that it inserts one zero pixel between neighboring image pixels, doubling the image size. The convolution kernel size in the transposed convolution layer is also 3×3 pixels.

The merge block is an image mosaic process. Once the result of the transposed convolution layer has been obtained, it is spliced into the feature image of the corresponding procedure in the contracting path. The principle of two convolution blocks is the same as the down-sampling operation, but it reduces the number of feature channel by half.

All the convolution kernel values are initialized with random numbers from a truncated Gaussian distribution and the values of biases are initialized as constant.

**Figure 3:**
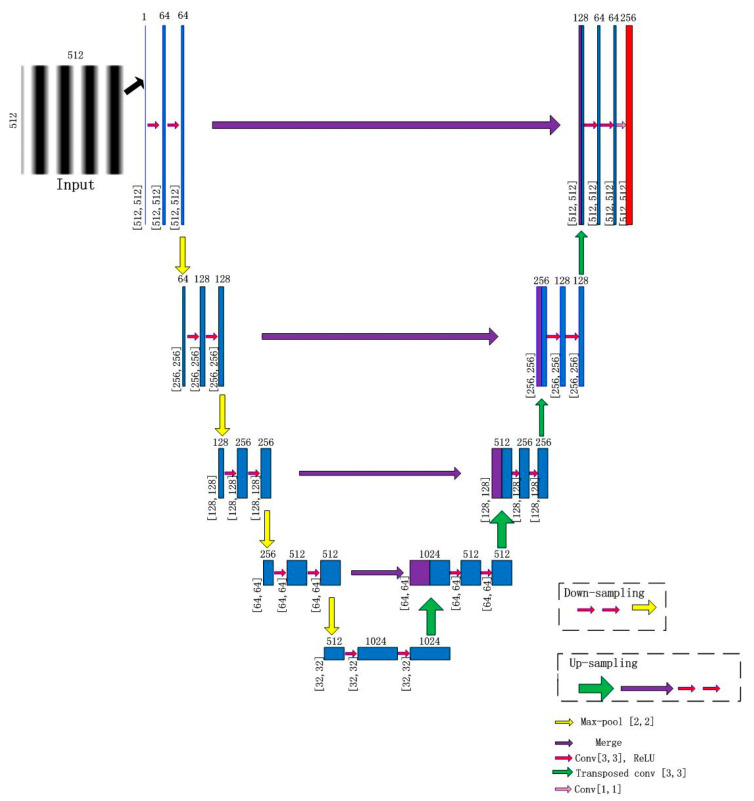
Process of network propagation.

The whole process of network propagation is shown in Figure 3. This process features a contracting path (left), a transition path and an expansive path (right). The size of the input fringe pattern is 512×512 pixels. After once down-sampling operation, the size of the image is changed to 256×256 pixels and the number of feature channels to 64. By repeating this process four times, the size of the image is reduced to 32×32 pixels and the number of feature channel changes to 512.

The transition path is consisted of two convolution blocks. Additionally, the feature channel doubles after two convolution blocks. The size of the image maintains 32×32 pixels and the number of feature channel changes to 1024.

Then, the result is subjected to the expansive path including four repeated up-sampling operation. The size of image is 64×64 pixels, 128×128 pixels, 256×256, and 512×512 pixels after each up-sampling when the numbers of feature channels are 512, 256, 128, and 64, respectively. Finally, a convolution operation is applied, and the size of the convolution kernel is 1×1 pixels. The size of the image maintains 512×512 pixels, and the number of feature channel changes to 256.

### 2.3. Network Training

Note that the warped phase results are periodic, when 1 output channel and MSE loss function are used, the result is easily restricted to the local optimal solution, where all output values tend to be 0. Thus, this problem is chosen to be converted into a classification problem. The result of the network is a three-dimensional matrix, and the size is 512, 512, 256 along x, y, and feature channel directions, respectively. For every pixel, the values along feature channel direction represent the possibility of being 0 to 255. A softmax function is used to reset the result to meet the requirement of probability distribution, so that cross-entropy [35] known as multi-class log loss can be used as loss function.

When the output of a pixel along the feature channel direction is $q_{1},q_{2},\cdots q_{n}$, the result of the softmax function can be described by

(3) $$\mathrm{soft}\max{\left(q\right)}_{i}=\frac{e^{q_{i}}}{\sum_{j=1}^{n}e^{q_{j}}},$$

where n represents the number of feature channels, and there is 256.

The probability distribution of reset q meets the following condition:

(4) $$\forall i;{\;q}_{i}\in \left[0,1\right];\overset{n}{\underset{i=1}{\sum }}q_{i}=1,$$

The ground truth g of each pixel supposed to be -pi to pi is scaled up to between 0 and 255. The probability distribution of training label p is given according to the formula:

(5) $$\forall i;{\;p}_{i=g}=1;{\;p}_{i\neq g}=0,$$

Cross-entropy is defined by [35]

(6) $$H\left(p,q\right)=-\overset{w}{\underset{x=1}{\sum }}\overset{h}{\underset{y=1}{\sum }}\overset{n}{\underset{i=1}{\sum }}p_{i}\left(x,y\right)\log q_{i}\left(x,y\right),$$

where p represents the training label and q represents the calculated result. The values of p and q are explained in the next section. n represents the number of feature channels, and w, h represents the width and height of the fringe pattern, respectively. The smaller the cross-entropy, the higher the probability that the actual and the calculated results are closer.

The backpropagation algorithm [36] is used to back-propagate the error into the network, and adaptive moment estimation (Adam) [37]-based optimization is used to optimize the weights of convolution kernels(w) and common basic terms(b) of all layers. An input is first propagated through the network. Then, the difference between the calculated and the desired output is backpropagated from the output layer to the first layer of the network, thereby adjusting the network weights in the opposite direction of the derivative of the network error with respect to each individual network weight. By repeating this procedure multiple times for each data in a training set, the network can be taught to map the inputs on the correct outputs. The batch size was 10, and the epoch was 1000. The learning rate was $10^{-4}$.

### 2.4. Output Principle

**Figure 4:**
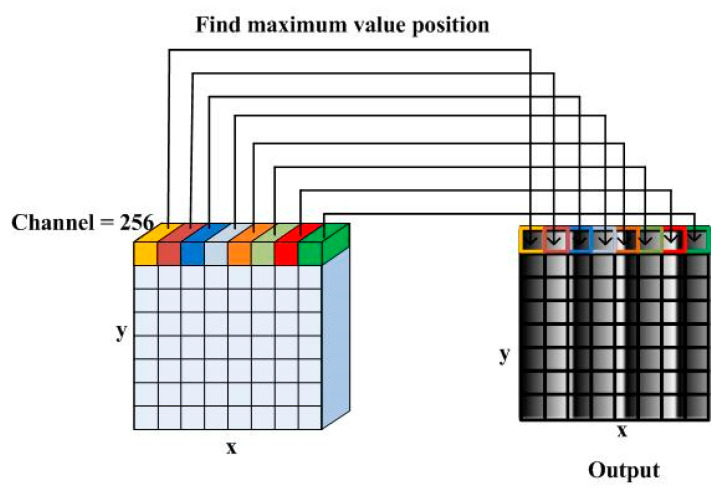
Output schematic.

The values of each pixel along feature channel direction represent the possibility of being 0 to 255. As Figure 4 shows, the output is the position corresponding to maximum possibility, so the output value is between 0 and 255. Note that the number of feature channels in the last convolution operation can choose more than 256, corresponding to higher resolution and more calculated time. At last, the output is restored to between -pi and pi.

## 3. Verification of Method

### 3.1. Simulation Image

Numerical simulations were carried out to test the performance of the proposed algorithm. From simple to complex, we used three equations to simulate fringe patterns, and the size of patterns was set to 512×512 pixels.

The gray level of the first kind of patterns was determined by Equation (7). A total of 1600 fringe patterns were obtained, in which the fringe number of a pattern was set between three and 44, and the fringe interval decreased gradually.

(7) I(t,x,y)=255×cos(((44×pi/512−3×pi/512)/1600×(t−1)+3×pi/512))×y),t=1,2,⋯1600;y=1,2,⋯512,

where t represents the series number of the pattern and y represents width in pixels.

The second and third kinds of fringe patterns were generated according to Equations (8) and (9). The 1600 fringe patterns with different fringe shapes were obtained through image cropping and rotation from a fringe pattern:

(8) I(x,y)=50+50×cos(peaks(1000)+20×pi/1000×y),y=1,2⋯1000,

(9) $$I\left(x,y\right)=50+50\times \cos(50-(\left(x-200{)}^{2}-\frac{(y-200{)}^{2}}{10,000})\right),\;x=1,2,\cdots 1000;y=1,2,\cdots 1000,$$

where x represents height in pixels and y represents width.

**Figure 5:**
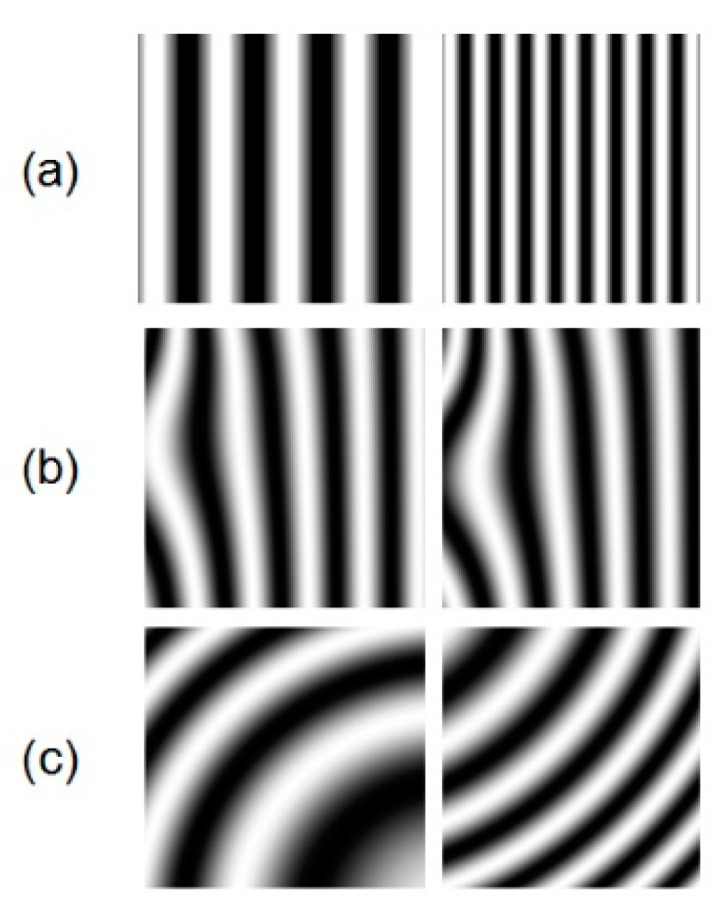
Simulated fringe patterns.

Figure 5 shows some simulation patterns. (a), (b), and (c) represent fringe patterns generated according to Equations (7)–(9), respectively. Of all simulation patterns, 100 fringe patterns were selected to evaluate the trained network and the rest were used to train the model. The gray level of the fringe patterns was set as the input to the network, and the wrapped phase data calculated by four-step phase-shifting method were set as the output.

### 3.2. Experimental Image

**Figure 6:**
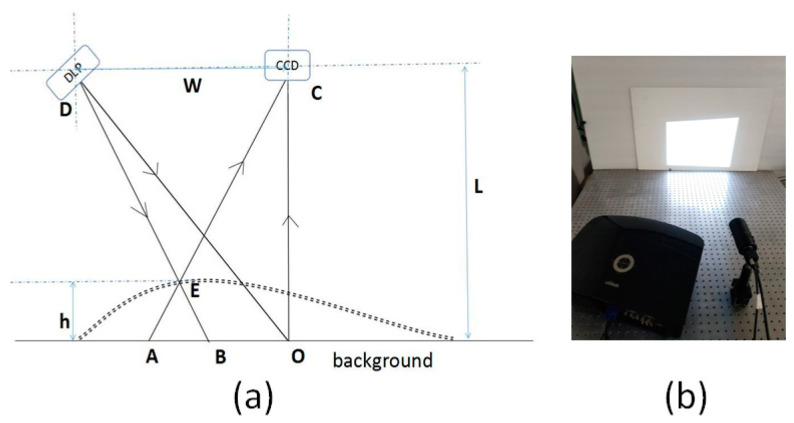
Fringe projection experiment: (**a**) Schematic layout; (**b**) physical diagram.

The fringe projection technique is often used to determine the 3D-profile of an object. When the fringe pattern is projected on a measured free surface, its phase is modulated by the height distribution of the object. We used the fringe pattern captured from the fringe projection experiment to verify the ability of the neural network to extract the phase map. Figure 6a,b show schematic layout and physical diagram of the experiment, respectively.

The optical path adopted oblique projection and vertical shooting. The digital fringe projector chosen was Vivitek-D5158HD at a resolution of 1920×1280 pixels. The camera used was the Basler ace 1600-20 g, with a resolution of 1600×1200 pixels. The optical axis of the projection and the receiving end intersected at point O. Moreover, the camera and the digital fringe projector were at the same height L. Due to modulation by the object’s height, light that was supposed to obtain at point B was cast on point E, but the light point recorded by the camera was A. Finally, the height information of the object was recorded in the fringe pattern.

**Figure 7:**
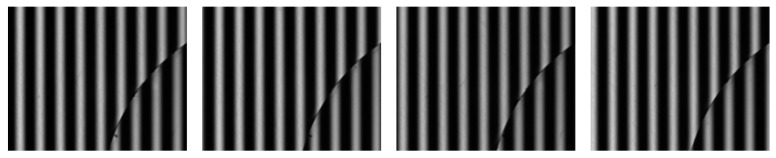
A fringe pattern from the disk experiment and its corresponding phase-shifting fringe patterns.

A 1-mm-thick disk was chosen as the measurement object. The position and angle of the disk were altered to obtain different fringe patterns. To obtain a sufficient number of images to meet the big data requirement of network training, such data extension as image cropping, translation, and rotation were used. One thousand fringe patterns were obtained. Figure 7 shows one of these and its corresponding phase-shifting fringe patterns.

**Figure 8:**
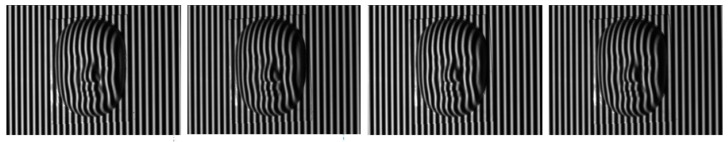
A fringe pattern from the mask experiment and its corresponding phase-shifting fringe patterns.

Another complex object, a facial mask from opera, was measured, as shown in Figure 8. The same operations were performed as before to yield another 1000 experimental patterns.

**Figure 9:**
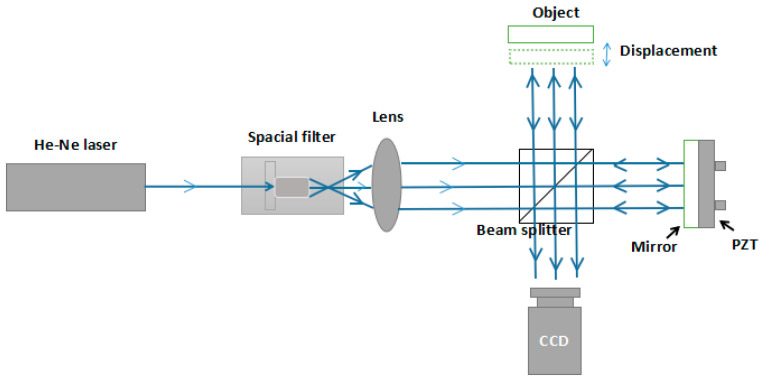
The schematic drawing of the measurement system.

**Figure 10:**
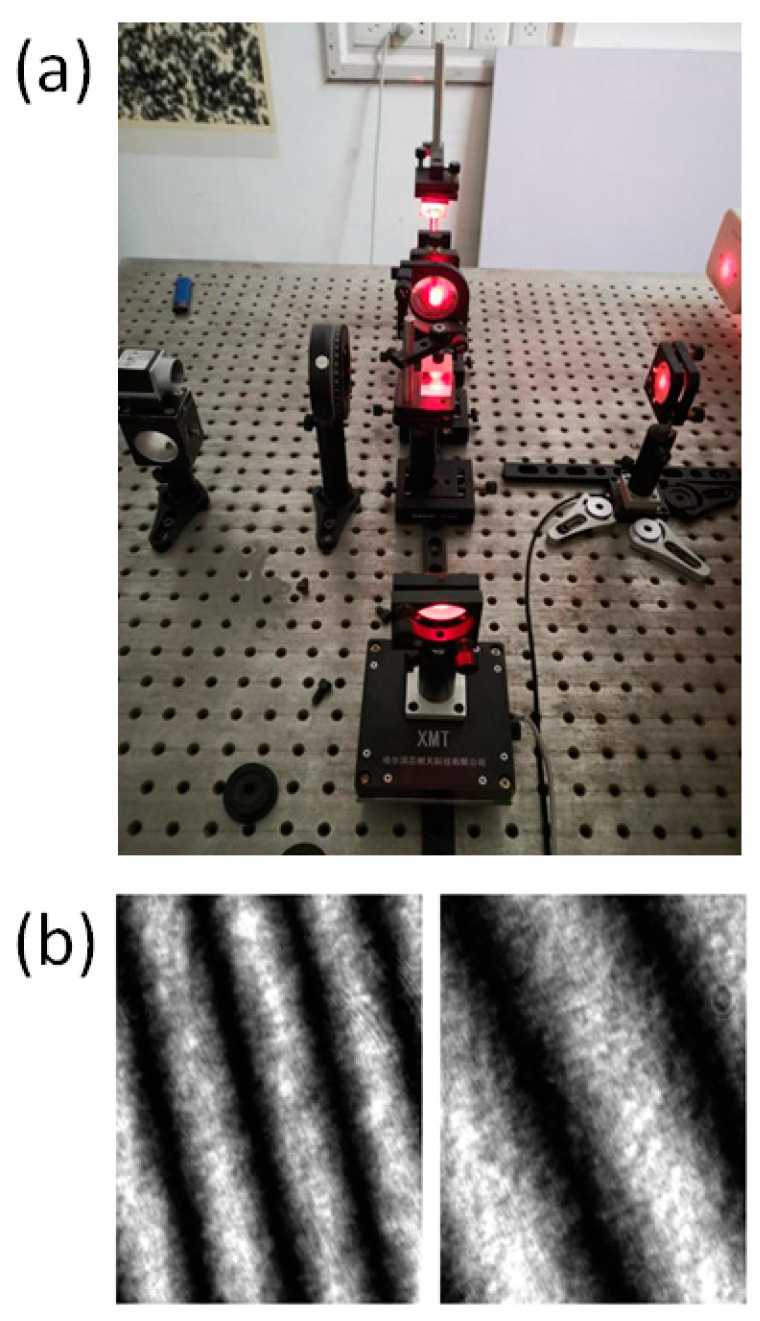
(**a**) Physical diagram. (**b**) Interferograms.

Michelson interferometry is widely used to measure out-of-place displacement. An MI-based measurement system was setup to obtain different interferograms, and Figure 9 shows a schematic drawing of the measurement system. A light beam was emitted from the He–Ne laser generator, and expanded as parallel light beams after going through a spatial filter and convex lens. The parallel light beams were then divided into two identical parts by a beam splitter (BS), and one each was introduced to the reference arm and the objective arm. In the objective arm, the light beam propagated onto the surface of the object and was reflected. The reference arm had a reflector coupled with a PZT used for phase-shifting. Finally, two reflected light beams were returned to the BS and interference onto the surface of a CCD. The phase of the captured interferogram recorded the out-of-place displacement information of the object. By changing the fringe interval or the position of the reflector, 100 interferograms were obtained directly, and the other 900 interferograms were obtained through data extension. Figure 10a,b show physical diagram and some interferograms, respectively.

The 100 fringe patterns from the fringe projection experiment and 100 interferograms were selected to evaluate the trained network, and the remainder was used to training the model. During the training, the wrapped phase dataset as the output of the neural network was calculated by the four-step phase-shifting method.

## 4. Results and Discussion

### 4.1. Computation Accuracy

#### 4.1.1. Results on Simulation Image

**Figure 11:**
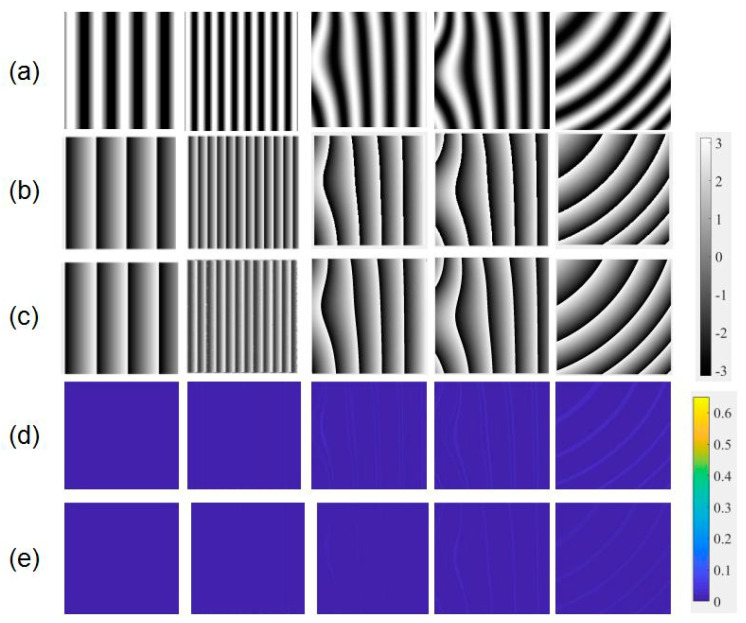
(**a**) Simulated fringe patterns, wrapped phase maps calculated from (**b**) four-step phase shifting method, and (**c**) neural network, and corresponding error maps from (**d**) wavelet transform method, and (**e**) neural network.

The 100 simulation fringe patterns were used to test the accuracy and robustness of the method. Figure 11 shows some simulated fringe patterns and corresponding calculated wrapped phase maps. Figure 11b was calculated by the four-step phase-shifting method and Figure 11c was obtained through the trained neural network. The results show that the two types of measurement aligned well, and patterns with different fringe intervals and types yielded the correct values. In order to further illustrate the accuracy of our proposed method, wavelet transform method was used to make comparison. The two-dimension wavelet transform was implemented according to approach proposed by Wang [38], and the window-modifying parameter chose to be 2. The error maps coming from different methods are shown in Figure 11d,e, respectively. The whole error level verified the high accuracy. 

To evaluate the accuracy of this method, we defined two types of error: systematic error (E) and standard deviation error (S) [31].

E is defined as

(10) $$E=\frac{1}{N}\frac{1}{M}\overset{M}{\underset{j=1}{\sum }}\overset{N}{\underset{i=1}{\sum }}\left|{d_{j}}_{i_{\mathrm{cal}}}-{d_{j}}_{i_{\mathrm{real}}}\right|,$$

where ${d_{j}}_{i_{\mathrm{real}}}$ represents the wrapped phase data of the i-th pixel in the j-th calculated image, and ${d_{j}}_{i_{\mathrm{cal}}}$ represents the calculated phase data of the i-th pixel in the j-th calculated image, which was also warped. M represents the number of calculated images, and N represents the number of pixels in an image. S reflects the average error, and its best possible score was zero.

S is defined as

(11) $$S=\sqrt{\left(\frac{1}{N}\frac{1}{M}\overset{M}{\underset{j=1}{\sum }}\overset{N}{\underset{i=1}{\sum }}{\left({d_{j}}_{i_{\mathrm{cal}}}-{d_{j}}_{i_{\mathrm{real}}}-E\right)}^{2}\right)},$$

where S reflects the deviation in the measured displacement corresponding to the mean value, and has is related to random error.

For the phase data of the simulation fringe patterns, the value of E was 0.03 rad and that of S was 0.07 rad.

#### 4.1.2. Results on Experimental Image

**Figure 12:**
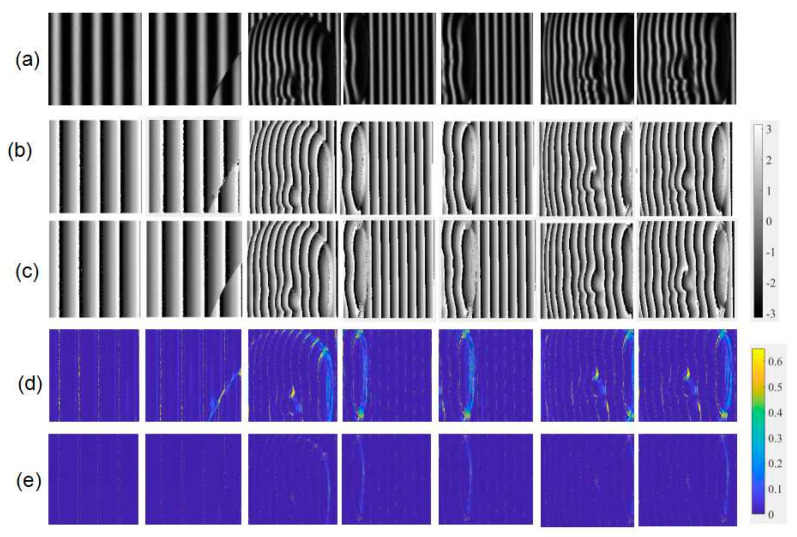
(**a**) Experimental fringe patterns, wrapped phase maps calculated from (**b**) four-step phase-shifting method, and (**c**) neural network, and corresponding error maps from (**d**) wavelet transform method, and (**e**) neural network.

**Figure 13:**
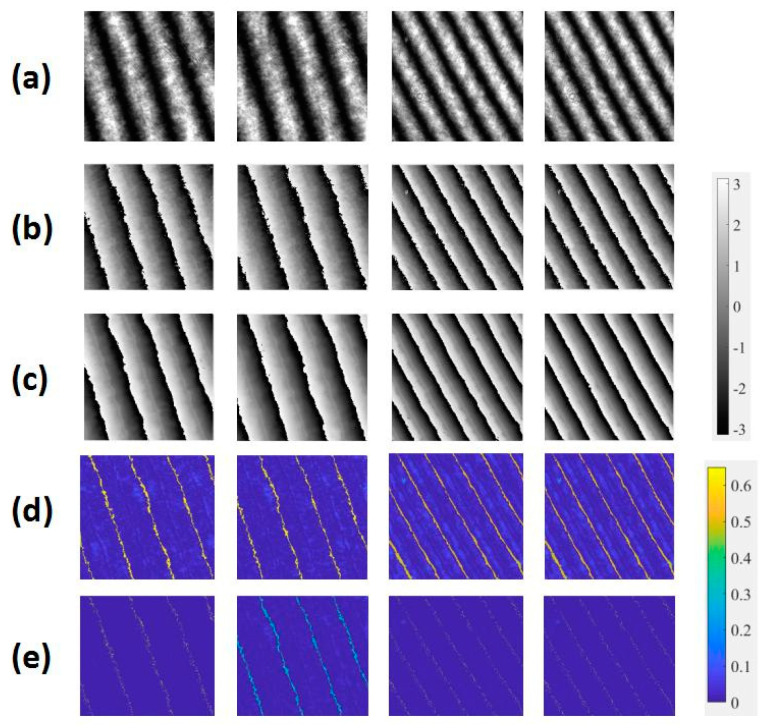
(**a**) Interferograms, wrapped maps calculated from (**b**) four-step phase-shifting method, and (**c**) neural network, and corresponding error maps from (**d**) wavelet transform method, and (**e**) neural network.

The 100 fringe patterns obtained from the experiment on fringe projection were used to test the accuracy and the robustness of the method. Figure 12 shows some patterns and their results of wrapped phase maps. The patterns in Figure 12a were chosen from different projection experiments that used an empty background, a disk, and different parts of a mask as measured object. One hundred interferograms were also used to test the accuracy and the robustness of the method. Some interferograms and their wrapped phase map results are shown in Figure 13. The results shown in Figure 12 and Figure 13b were calculated by the four-step phase-shifting method, and those shown in Figure 12 and Figure 13c were obtained through the trained neural network. Through the comparison, we see that the results of the two methods were consistent. They also show that both experimental fringe projection patterns and interferograms yielded the correct results, and verified the robustness of the trained neural network and the feasibility of the machine learning method. Additionally, error maps are presented in Figure 12d,e and Figure 13d,e. Through contrast, errors of our proposed method were reduced obviously, demonstrating its improved performance in measuring complex objects under environmental noise. Thus, the proposed method owns higher noise resistance ability compared with wavelet transform method. 

By referring again to the results of calculation of the four-step phase-shifting method, we used E and S to evaluate the accuracy of the experimentally obtained fringe pattern. For the wrapped phase data of the experimental fringe patterns, the value of E was 0.10 rad and S was 0.08 rad. For the wrapped phase data of interferograms, the value of E was 0.22 rad and S was 0.24 rad. The results verified the precision of this trained neural network and the accuracy of the learning-based method, as well as the system’s ability to resist environmental noise. We also see that with decreasing quality of the fringe pattern, error increased.

### 4.2. Computation Efficiency

In order to illustrate the computation efficiency of the proposed method, the wavelet transform method was used to make comparison, which also only needed a single fringe pattern. The wavelet transform method was also programming using python language. 10 simulation patterns, 10 fringe projection patterns and 10 interferograms were calculated, respectively, and the values of E and S were shown in Table 1. The average calculated time of one pattern were recorded in Table 2.

From Table 2, we can see, once the neural network was determined, the calculated time of the neural network method have no matter with the fringe pattern quality, only depending on the network structure and the input size. Under the same accuracy, the calculated speed of the proposed method is 20 times faster than the wavelet transform method.

### 4.3. Discussion

**Figure 14:**
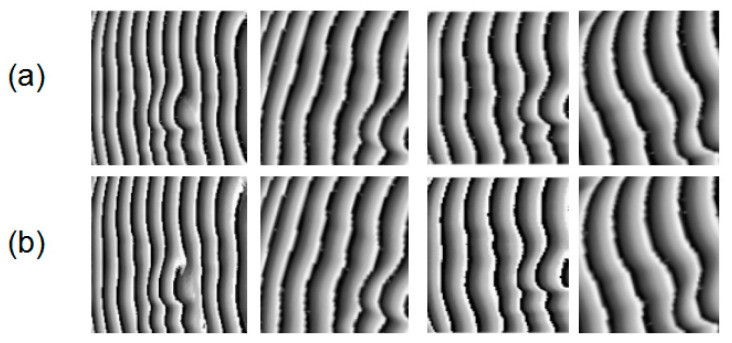
Calculated wrapped phase maps from (**a**) four-step phase-shifting method, and (**b**) neural network.

From Figure 12c, we find that different measured objects can yield correct results regardless of the change in the position or the angles of objects. Figure 13c shows that this trained neural network can be applied to interferograms with lower pattern quality. To verify the network’s ability to handle more complex fringe patterns with different shapes and intervals of fringes, some fringe obtained from such data extensions as image extension and rotation were calculated, and consistent results were obtained as shown in Figure 14. The results of Figure 14 further verify the robustness of this trained neural network.

This learning-based method is an improving process. The more different fringe patterns are learned, the more complex model can be calculated. A sufficient amount of big data can support this neural network to adapt to all kinds of fringe patterns. Different fringe patterns verified its precision with the four-step phase-shifting method, however, this end-to-end neural network only needs one original fringe pattern to obtain the corresponding wrapped phase map.

This method requires a considerable amount of training data, which is time-consuming in data preparation. This training can be a continuous process. Once a batch of fringe patterns has been trained, the obtained neural network can be saved. The next batch of images can then be trained on the saved neural network, which can significantly reduce the time needed for training.

## 5. Conclusions

In this paper, we proposed a fringe wrapped-phase map extraction method based on the U-net neural network that can obtain the wrapped phase map directly from a fringe pattern. The results of simulated and experimental fringe patterns verified the efficiency and the robustness of this method. At the same accuracy, the proposed method boasts easy operation and a simple principle compared with the traditional phase-shifting method and owns faster speed than wavelet transform method.

## Figures and Tables

**Table 1:** E and S of the proposed method and wavelet transform method.

Value	E(rad)			S(rad)		
Patterns	Simulation	Fringe Projection	Interferograms	Simulation	Fringe Projection	Interferograms
The proposed method	0.03	0.10	0.22	0.07	0.08	0.24
Wavelet transform method	0.05	0.15	0.24	0.08	0.14	0.29

**Table 2:** Calculated time of the proposed method and wavelet transform method.

Time (s)	Simulation Patterns	Fringe Projection Patterns	Interferograms
The proposed method	0.069	0.066	0.071
Wavelet transform method	1.154	3.152	2.850

## Data Availability

Not applicable.
